# Scalp-Wound, Followed by Purulent Arachnitis—Trephining—Death—Autopsy

**Published:** 1857-02

**Authors:** Geo. Amerman

**Affiliations:** Late House Surgeon to Bellevue Hospital


ARTICLE II.—*Scalp Wound, followed by Purulent Arachnitis—Trephining—Death—Autopsy.* By GEO. AMERMAN, M.D. late House Surgeon to Bellevue Hospital.

George Roach, aged twenty-one, born in New York, by occupation a machinist. He was admitted into Bellevue Hospital on Friday, May 2d, 1856. The day previous to admission he received a blow on the head with a cleaver, which produced a scalp-wound two inches in length. Its anterior extremity was situated three and a half inches above the supra orbital ridge, and half an inch to the left of the mesial line. It extended backward in an oblique direction, its posterior extremity being over the sagital suture. The soft parts were completely divided, leaving the bone and pericranium apparently uninjured and healthy.

He was a robust man, of intemperate habits, but denied ever having had syphilis. His appetite and strength were good. He was about the ward and yard for two days after admission; and, when asked how he felt, replied, 'Very well.' On Monday, May 5th (third day after admission), the left side of his face became swollen, hot, and painful. The slightest pressure over the body or ramus of the lower jaw on that side produced severe pain. Leeches and warm fomentations were used with free purgation.

On Wednesday (fifth day after admission), the acute pain and increased heat had entirely disappeared; however, there remained some tension of the part and an obscure fluctuation. An opening was made from the inside, commencing the incision at the margin of the alveolar processes and keeping close to the jaw, extending it downward to near its lower border. About one drachm of pus was discharged; and on introducing a probe it was found that nearly the whole of the left half of the inf. max. was carious. A poultice was then applied, and subsequently a counter-opening made which afforded complete relief. The internal opening remained patent, and the teeth of that side fell out. Pus of an unhealthy character exuded from these openings. The wound of the scalp remained open and showed no evidences of healing; the few granulations about its edges were weak and flabby; the cranium denuded and carious. His general condition continued good until the 12th May (eleventh day after admission, when he began to complain of weakness, loss of appetite, a curious sensation about the head (which he was unable to describe), and sleeplessness.

On the 18th he became petulant and impatient, eyes staring, answers brief and ill-humored, and manner hasty and undecided.

*May 19th*—He was restless last night, and got up several times to walk about the ward. Pulse 84; resp. 26; skin warm and moist; tongue slightly coated with a white film; eyes more natural; pupils normal; no tendency to coma; no paralysis.

*Seven o'Clock, P. M.*—Intense chemosis of both eyes. Patient irrational. When asked a question, he either repeats it or gives an answer entirely disconnected with the inquiry. Bowels not moved since day before yesterday. Ordered a cathartic of ol ricini.

*May 20th*—Pulse 74; resp. 24. When undisturbed he lies as if he were quietly asleep. If the eyes or wound are rudely handled, he resists; if spoken to is easily aroused, but cannot be made to answer questions. Chemosis extreme, the conjunctiva projecting between the lids. Pupils normal, and act well. No paralysis. Bowels still costive. Ordered an enema of ol terebinth and ol ricini.

*May 21st*—Pulse 80; resp. 28. He was delirious the greater part of last night. Bowels moved freely after the injection, with a free passage of urine. He is now semi-comatose, and incapable of being aroused, except on very rude handling. If asked to put out his tongue he seems to understand what is said to him, and makes a feeble effort to obey. Skin hot and dry. No increase in the temperature of the head. Pupils normal, and act promptly under the stimulus of a strong light. Chemosis same as at last note. Occasionally, in expiration, there is a slight puffiness of the lips and cheeks; there is, also, COMPLETE paralysis of the right side. At 10½ and again at 11 o'clock A.M. he had a convulsion. They began on the right side, and lasted from one-half to one minute. They very much resembled a paroxysm of epilepsy, except there was no foaming at the mouth, and no unusual drowsiness following them.

*Half-past Two, P.M.*—Pulse 108; resp. 36. He has had several convulsions during the day, but his condition has not materially changed for the last two hours. It was now decided to apply the trephine, on the supposition that matter had formed on the left side between the dura mater and skull. The original wound was lengthened to the extent of three inches, and a transverse one of one and three-fourths made to cross it at right angles. The pericranium was detached, and the instrument placed on the carious part of the bone, a little to the left of the mesial line. On sawing through the external table, a small quantity of pus escaped from the diploe. On entire removal of the disc, a few drops only of pus was discharged. The dura mater was of a brownish hue, roughened, now pulsating and slightly protruding through the cranial aperture. A longitudinal incision, made with a thumb-lancet, completed the operation. About two drachms of pus was discharged. There was some venous hemorrhage from the internal veins of the skull; otherwise the bleeding was slight. After the operation, the flaps were replaced, and cold-water dressing applied over the part. Pus and blood continued oozing from the wound. Pulse 80; resp. 30; skin warm and moist; stupor unchanged, being still insensible and incapable of being aroused; paralysis of right leg ENTIRELY gone; right arm *very much less*, but not completely removed.

Ordered an enema to be given immediately. At 8 o'clock the convulsion returned. He had one at 8, a second at 8½, and a third at 9 o'clock, which I saw. It lasted about one-half a minute, and chiefly affected the right side. His respiration immediately *before* and *after* each attack, was labored and irregular. Ordered the cold-water dressing discontinued. The enema produced a free movement of the bowels.

*Twelve o'Clock, Midnight*—Pulse 80; resp. 30. No recurrence of the convulsions since last note.

*May* 22*d*, *Seven o'Clock*, *A.M.*—At 2 o'clock this morning the convulsions returned, coming on at short intervals until 6 o'clock, during which time he had five paroxysms (four slight and one severe). Wound still discharges pus. The muscles of the tongue and right side of the face are in constant spasmodic action. Chemosis considerably less than yesterday. Consciousness somewhat improved. Pupils natural. Pulse 78.

*Seven o'Clock*, *P.M.*—Very little change since morning. During the day he has had two convulsions. No paralysis. Pulse 80.

*May* 23*d*, *Eight o'Clock*, *A.M.*—Patient spent a quiet night, with the exception of having had three convulsions. The same twitching of the tongue and right side of face as yesterday. Swelling of the eyes less; pupils and skin normal. Occasionally the muscles of the larynx act spasmodically, producing irregular respiratory movements; it also occasionally happens that the right arm and leg are affected in the same way. He urinated this morning when told to, and also drank some milk. Wound discharging pus.

*Eight o'Clock, P.M.*—He has had, during the day, several severe convulsions, and is now wholly insensible and incapable of being aroused. Since the previous note he has rapidly declined, and is almost worn out from repeated attacks of convulsions. There is again *complete* paralysis of right side. Respiration free and easy. Pupils natural and act promptly.

*May 24th*—He died at 7 o'clock this morning. I learned, from the Orderly of the ward, that during the night his convulsions were almost constant. The last came on about fifteen minutes before he died—after it, respiration was free and easy.

*Autopsy, Twelve hours after Death*—Weather warm. No rigor mortis. Lower jaw not examined. On removing the calvarium, the dura mater about the site of the injury presented a purplish-gray appearance. On section of it, about one-half an ounce of pus was discharged. The whole of the convex surface of the left hemisphere was covered with a thick layer of pus and false membrane. The pus extended down between the sulci. The membrane was dense, and could be raised and peeled off quite easily. There were some white opaline spots and lines following the course of the vessels of the opposite side, but no pus or distinct fibrinous effusion. The ventricles contained the usual amount of serum, and presented no trace of disease. The choroid plexus, the corpora striata, and the optic thalami, presented a normal appearance. Cerebellum natural. Thoracic and abdominal organs healthy.

### REMARKS.

If we, in brief, review the most prominent symptoms of this case, we shall see how well they indicated the actual changes occurring in the brain and its coverings. Our patient was a man in good health, with no constitutional or acquired predisposition, and hence we may reasonably suppose the wound proved fatal from its own importance. On the 1st of May he received the blow. It produced no other effect than a simple scalp-wound, two inches in length. He came under our charge the next day, and we deemed the injury of so trivial a nature that no treatment, except simply cleansing the part, was thought necessary. (The affection of the jaw does not seem to us to be at all connected with the progress or result of the case. We regard it as being an accidental circumstance, occurring during the time he was suffering from the injury. It is, however, interesting, as there was no evidence of disease in the jaw until the third day after his admission—nor were we able to obtain a history of any previous affection—when we find him suddenly attacked with symptoms of an acute inflammatory nature, and two days afterward caries of almost the entire half of the lower jaw).

He remained under close observation for eleven days, during which time there was not a single unfavorable or suspicious cerebral symptom, and even then the only indication of trouble was his restlessness, anorexia, and an indescribable feeling about the head, all of which might have been due to the accidental affection of the jaw.

On the morning of the 19th (twentieth day after admission), his pulse is 84, skin moist, and pupils natural. At 7 o'clock P.M. of the same day, we have intense chemosis of both eyes, and patient irrational—decided symptoms of cerebral trouble. He grew rapidly worse until 2½ o'clock P.M. of the 21st, when we find him *completely* paralyzed on the right ride, suffering from frequent convulsions, incapable of being aroused, skin hot and dry, pulse 108, resp. 36. The order of marked cerebral disturbance, was, *first*, delirium; *second*, paralysis; *third*, convulsions. The *delirium* was at first of a transient nature, the patient being disposed to wander about the ward; subsequently, it became of that troublesome and busy character seen not infrequently in delirium tremens; but at no time was he at all furious or wild. The *paralysis* was hemiplegic, affecting only the right side, and was complete, sensation as well as motion being entirely lost.

The *convulsions* were epileptiform in character, and began on the right side. They lasted about one minute, the muscles of both sides becoming, finally, affected alike in one general spasmodic action.

If, now, we compare these symptoms with what we conceive to have been the actual changes occurring in the brain and its coverings, we shall see that so far as we have yet studied our case they coincide exactly with our theories of the supposed effects of cerebral meningitis.

*First*, we had our prodromes or stage of congestion, marked by sleeplessness, anorexia, and a curious sensation about the head.

*Second*—Our stage of irritation and inflammation, marked by a staring eye and delirium.

*Third*—Our stage of effusion, marked by paralysis and convulsions.

We now trephine him—and let us contrast the symptoms that preceded with those that follow the operation:—His pulse has fallen from 108 to 80; resp. from 36 to 30. Paralysis of right leg *entirely* gone; right arm *very much less*, but not completely removed. Convulsions for a time (seven hours) absent. Delirium, of a low, muttering character. Stupor unchanged. Pupils and skin normal. We notice, then, that nearly all the severe symptoms were relieved. His improvement was immediate, and sufficiently apparent to have justified the operation. For a time there seemed to be some good ground for a hope that he might survive, but unfortunately it proved transient, for seven hours subsequently we find him again convulsed, and fifty-four hours after *completely* paralyzed on the right side. His condition, from the recurrence of the convulsions, grew rapidly worse, and he died sixty-five hours after the operation, apparently from exhaustion.

We regard the relief afforded by the operation, due to the escape of pus, which must have produced pressure sufficient to cause paralysis, and which could scarce exist without such a degree of irritation as to provoke a disturbed cerebral action and consequent irregular muscular movements. The recurrence of these grave symptoms and final result of the case, were evidently due to the extension of the disease, and re-accumulation of pus in the arachnoid. He died, worn out from the severe and oft-repeated convulsions.

After death we find the post mortem appearances of a well-marked case of 'purulent arachnitis.' It seems to us to be a classical case of the above affection, and one in which the symptoms throughout were well-marked, and hence we deemed it worthy of publication. One important practical question suggests itself to our mind, and when we have briefly called attention to it, we leave the case to speculative physiologists, who cannot fail to perceive in it several phenomena of curious import. The meningitis and fatal termination of the case, were undoubtedly due to the scalp-wound—a simple lacerated wound, extending through the soft parts, leaving the bone and pericranium uninjured. Such wounds are of common occurrence in the practice of every surgeon; and if they are not infrequently followed by cerebral affection and death, it becomes a question of great importance that we should be able to determine, in some way, *when* such complications are most likely to supervene, and what are the *first evidences* of their occurrence. During our residence in Bellevue Hospital, we had several cases of scalp-wounds in charge, but the above was the only one followed by secondary trouble. They all recovered under simple treatment, and were discharged cured in from six to twenty days. In some of these cases there were abscesses, puffy tumors, and purulent collections between the bone and pericranium, with superficial caries; but even under such circumstances they invariably got well. The only departure from the general course of such wounds noticed in the above case, previous to the supervention of decided cerebral difficulty, was its external appearance. It at no time presented a healthy granulating surface. The few granulations present were weak, large, and flabby. The pericranium became *early* detached, and the bone carious. We think some writers have placed considerable stress on the importance of the character of the wound, and from the above case we are led to agree with them in considering it an unfavorable symptom, and one that ought to place us on our guard and make us vigilant in detecting the first indication of trouble. Detachment of the pericranium and caries of the bone occurring *early*, must be regarded as unfavorable; but there are, doubtless, many such cases without any other trouble than lengthening the time required for a complete cure. The unexpected length of our paper forbids further consideration of this subject, and if we have succeeded in directing attention to this class of injuries we shall feel amply rewarded for our time in collecting the notes of the case.

